# Supplementary figures and images for: Nested PCR Detection of Pythium sp. from Formalin-Fixed, Paraffin-Embedded Canine Tissue Sections
Source: Vet Sci. 2022 Aug 19;9(8):444. doi: 10.3390/vetsci9080444 (PMC9412607; doi:10.3390/vetsci9080444)

Original figures from Gels:

**Figure S1**

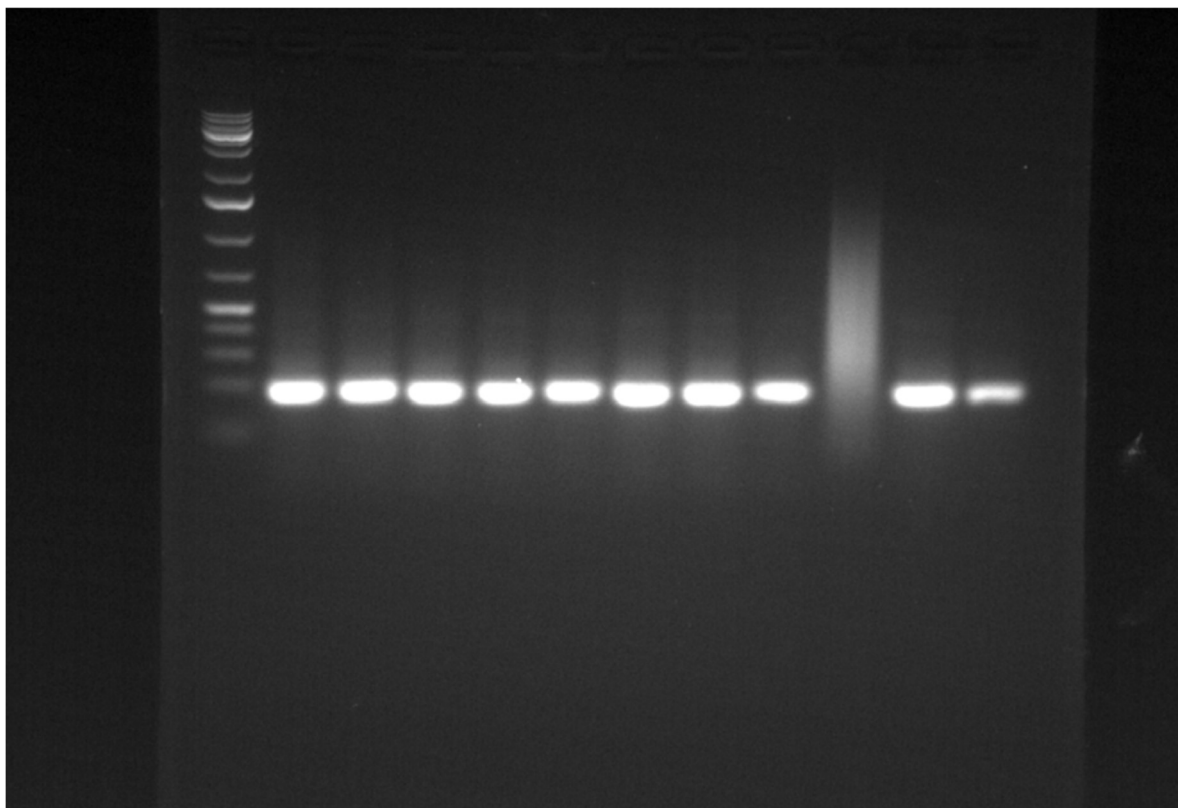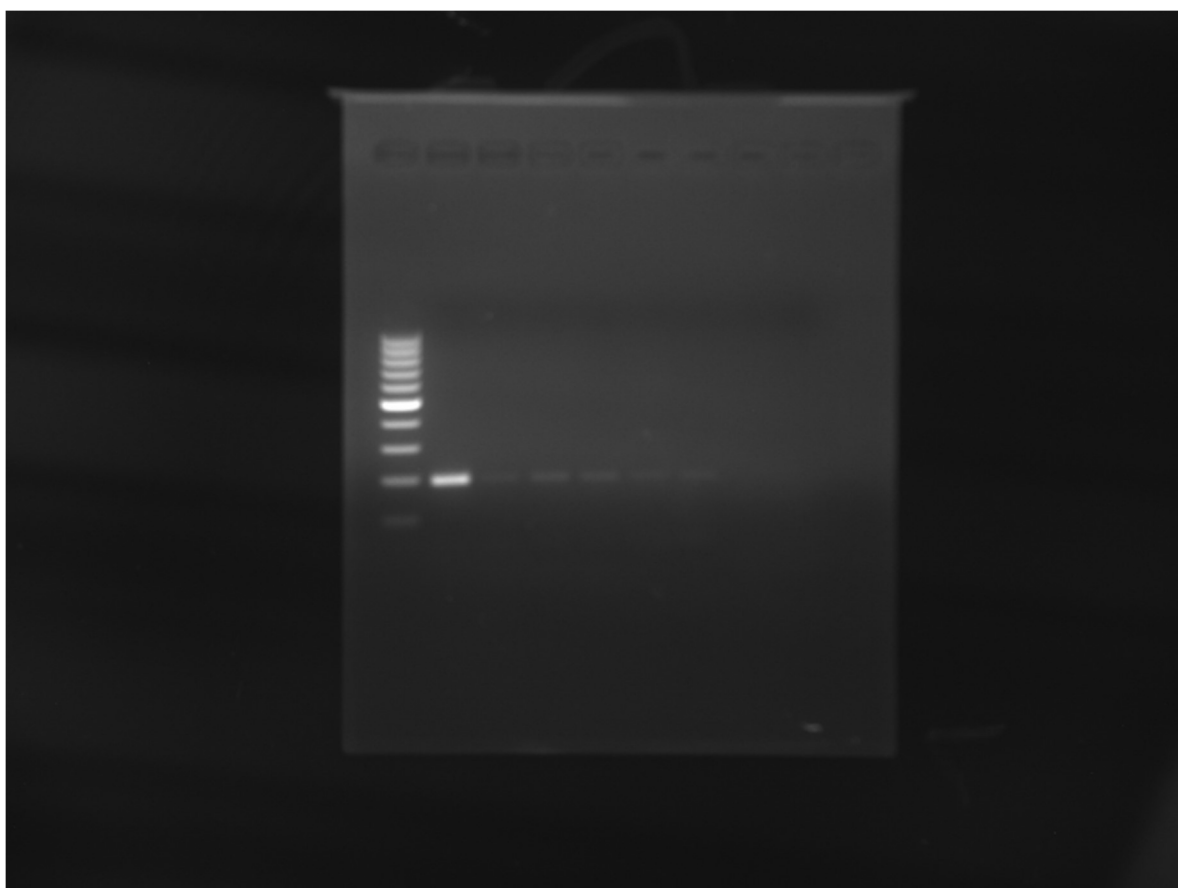

Supplement: Supplementary file 1 [file vetsci-09-00444-s001.zip › vetsci-1786520-Supplementary.pdf]
